# Anisotropic nonlinear optical responses of Ta_2_NiS_5_ flake towards ultrafast logic gates and secure all-optical information transmission

**DOI:** 10.1515/nanoph-2024-0404

**Published:** 2024-10-23

**Authors:** Lei Yan, Ziyao Gong, Qinyong He, Dechao Shen, Anping Ge, Ye Dai, Guohong Ma, Liaoxin Sun, Saifeng Zhang

**Affiliations:** Department of Physics, 34747 Shanghai University, Shanghai 200444, China; State Key Laboratory of Infrared Physics, Shanghai Institute of Technical Physics, Chinese Academy of Sciences, Shanghai 200083, China; University of Chinese Academy of Sciences, Beijing 100049, China

**Keywords:** saturable absorption, excited state absorption, carrier dynamic, all-optical logic gate, optical information transmission

## Abstract

Optical logic gates based on nonlinear optical property of material with ultrafast response speed and excellent computational processing power can break the performance bottleneck of electronic transistors. As one of the layered 2D materials, Ta_2_NiS_5_ exhibits high anisotropic mobility, exotic electrical response, and intriguing optical properties. Due to the low-symmetrical crystal structures, it possesses in-plane anisotropic physical properties. The optical absorption information of Ta_2_NiS_5_ is investigated by anisotropic linear absorption spectra, femtosecond laser intensity scanning (*I*-scan), and non-degenerate pump-probe technology. The *I*-scan results show a distinct maximum of ∼4.9 % saturable absorption (SA) and ∼4 % reverse saturable absorption (RSA) at different polarization directions of the incident laser. And, these unique nonlinear optical (NLO) properties originate from the anisotropic optical transition probability. Furthermore, the novel Ta_2_NiS_5_-based all-optical logic gates are proposed by manipulating the NLO absorption processes. And, the all-optical OR and NOR logic gates possess an ultrafast response speed approaching 1.7 THz. Meanwhile, an all-optical information transmission method with higher security and accuracy is achieved, which has promising potential to avoid the disclosure of information. This work provides a new path for designing versatile and novel optical applications based on Ta_2_NiS_5_ materials.

## 1 Introduction

The logic gates used to perform Boolean logic operations are basic and important parts of computation and information processing [1], [2]. However, the performances of current logic gates based on electronic transistors are approaching limitations because of the excessive energy consumption of switching logic states and operation speed limiting by electronic physical devices [1], [3], [4], [5], [6]. This calls for a novel strategy that satisfies the demands of ultrafast operating speed and processing growing information. The all-optical logic gate operates by optical nonlinearity utilizing photons as information carriers with greater speeds and can process a huge amount of information [1], [7], [8], [9], [10], [11]. In high-capacity optical networks, all-optical logic gates will play an essential role in performing the optical signal processing and improving operating speed to surpass the limitations of electronics. Therefore, it is primarily necessary to explore novel materials with superior nonlinear optical (NLO) properties and ultrafast response speed.

Ternary chalcogenide, a new type of layered semiconductor material with weak van der Waals forces between layers, are non-toxic, easy to synthesize and exfoliate [12], [13], [14]. They have recently attracted wide attention in photocatalysts, photodetector, energy storage devices and other fields [15], [16], [17], [18], because of their unique structures, and remarkable physical properties. Compared to single or binary elements materials [19], [20], the added new degree of freedom for ternary chalcogenides, which can be utilized to tune the electrical and optical properties by changing the compositions, expands the opportunities for designing valuable and functional devices [21], [22], [23]. For example, the bandgap can be flexibly tuned by manipulating the ratio of Cu/In in copper-indium-selenium system resulting in novel properties or functionalities [24]. And, the CuFeS_2_ nanosheets with robustly active exhibit higher performance for hydrogen evolution reaction, when compared to their binary compounds (CuS and FeS_2_) [25]. As an interesting member of ternary chalcogenide, Ta_2_NiS_5_ has low-symmetrical crystal structures with anisotropic physical properties [13], [26], [27]. Meanwhile, monolayer and bulk Ta_2_NiS_5_ are both direct bandgap semiconductors with a bandgap of 0.39 and 0.36 eV, respectively [26]. Due to the superior optical, electrical, and thermal properties, it has been widely utilized in biosensors, photoacoustic theranostics, and saturable absorbers [13], [26], [28], [29], [30], [31], [32]. Yan et al. measured the saturable absorption SA behavior of the Ta_2_NiS_5_ and realized a passively Q-switched laser [32]. And, layered Ta_2_NiS_5_ has been used as photoconductive detector presenting a fast, endurable, and anisotropic photoresponse [13]. The response time and modulation depth are key parameters for ultrafast devices, like optical logic gates and all-optical switches, which can be characterized by anisotropic NLO absorption and carrier dynamics, respectively. To our knowledge, these properties of Ta_2_NiS_5_ have not been studied.

In this work, we have presented a comprehensive study of the in-plane anisotropic optical properties of Ta_2_NiS_5_ flake. The polarized Raman spectroscopy was used to identify the armchair and zigzag directions of the Ta_2_NiS_5_ flake. And, the NLO absorption and carrier dynamics of Ta_2_NiS_5_ flake were investigated by polarization-dependent intensity scanning (*I*-scan) and pump-probe systems, respectively. The diverse nonlinear responses of SA and excited state absorption (ESA) were observed due to the different transition probability, which can be flexibly switched by manipulating the polarization of the input laser. By fitting the experiment results, the NLO parameters and carrier relaxation time were obtained. Utilizing the unique NLO absorption, we have demonstrated the Ta_2_NiS_5_-based all-optical OR and NOR logic gates with ultrafast response speed of ∼1.7 THz. In addition, an accurately secure information transmission method encoded by intensity and polarization of incident laser has been achieved. These results provide new perspective for exploring ultrafast photonic devices, and facilitate effective and versatile Ta_2_NiS_5_-based applications.

## 2 Results and discussion

**Figure 1:**
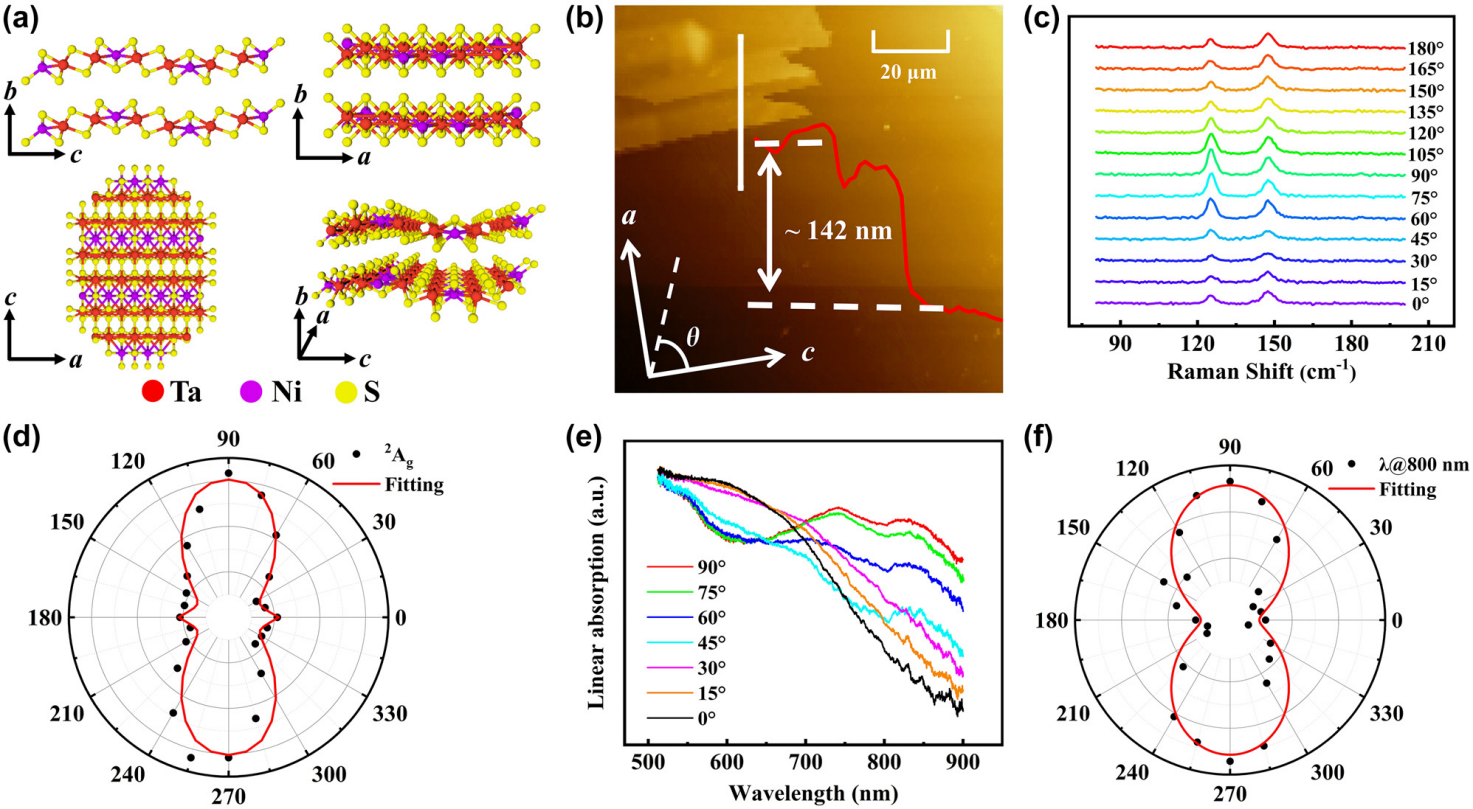
The structure and characterization of Ta_2_NiS_5_. (a) Schematics of the layered Ta_2_NiS_5_ crystal structure. (b) AFM image and the thickness of the Ta_2_NiS_5_ flake. (c) Typical polarized Raman spectra of the Ta_2_NiS_5_ flake measured by 532 nm laser with different polarization angles. (d) The anisotropic Raman intensity of ^2^A_g_ mode. The black dots and red curve are experimental data and fitting result, respectively. (e) Polarization-dependent linear absorption spectra from 0° to 90°. (f) Polar plot of the linear absorption at 800 nm.

Ta_2_NiS_5_ is a novel ternary chalcogenide with van der Waals layered structure [33], [34], and the crystal structure is shown in Figure 1(a). The adjacent Ta_2_NiS_5_ layers are stacked along the *b* axis by van der Waals forces and can be easily exfoliated. The Ni and Ta atoms located in the middle sheets connect with S atoms in the top and bottom sheets forming tetrahedral and octahedral structures (NiS_4_ and TaS_6_ units) along the *c* axis [26], [27], respectively. Because of the different structures in *a* axis (armchair direction) and *c* axis (zigzag direction), Ta_2_NiS_5_ considerably possesses in-plane anisotropic properties. The armchair and zigzag directions of the Ta_2_NiS_5_ flake are determined by the ARPRS. The thickness of Ta_2_NiS_5_ flake measured by atomic force microscopy (AFM) is about 142 nm in this study, as shown in Figure 1(b).

The polarized Raman spectroscopy is employed to identify the crystal orientation. In Figure 1(c), it can be seen that two Raman peaks are found at 127.0 and 148.6 cm^−1^, corresponding to the ^2^A_g_ and ^3^A_g_ modes [13], [26]. The excitation laser propagates along *b* axis and the polarization direction is controlled through a half-wave plate. To demonstrate the anisotropy of the Raman intensity, the polar plot of ^2^A_g_ mode is drawn in Figure 1(d). The intensity of Raman modes can be expressed as [35],

(1) $$I\propto {\left|e_{i}Re_{s}\right|}^{2}$$

where ***e***_***i***_ and ***e***_***s***_ are the unit polarization vectors of input and scattered laser, respectively, and ***R*** is the Raman tensor. The A_g_ mode of Raman tensor is given by [36],

(2) $$R\left({\text{A}}_{\text{g}}\right)=\left(\begin{array}{ccc} \left|a\right|{\text{e}}^{\text{i}\varphi_{a}} & 0 & 0\\ 0 & \left|b\right|{\text{e}}^{\text{i}\varphi_{b}} & 0\\ 0 & 0 & \left|c\right|{\text{e}}^{\text{i}\varphi_{c}} \end{array}\right)$$

where *a*, *b*, and *c* are the amplitude, *φ*_*a*_, *φ*_*b*_, and *φ*_*c*_ are the phases of Raman tensor elements [37], [38]. Thus, the anisotropic Raman scattering intensities can be derived as,

(3) $$\begin{array}{ll} I\left({\text{A}}_{\text{g}}\right) & \propto {\left|c\right|}^{2}\left\{{\left(\text{si}{\text{n}}^{2}\theta +\frac{\left|a\right|}{\left|c\right|}\cos\varphi_{ca}\text{co}{\text{s}}^{2}\theta \right)}^{2}\right.\\ & \;+\left.\left.{\left(\frac{\left|a\right|}{\left|c\right|}\sin\varphi_{ca}\text{co}{\text{s}}^{2}\theta \right)}^{2}\right\}\right. \end{array}$$

where *φ*_*ca*_ = *φ*_*c*_ − *φ*_*a*_. The polarization-dependent Raman intensity can be properly fitted as shown in Figure 1(d). It has the strongest intensity when the polarization angle of incident laser at 90° or 270°, and shows a 180° periodic variation. The Raman intensities are dependent on crystal direction and can be used to identify the crystal axis of Ta_2_NiS_5_. Meanwhile, the A_g_ modes reach the global maximum along the *a* axis (armchair direction) and local maximum along the *c* axis (zigzag direction) [13]. In our study, the similar pattern can be seen in Figure 1(d) of ^2^A_g_ mode. It indicates that the *θ* is the angle between the polarization direction of input laser and the *c* axis of the Ta_2_NiS_5_, as shown in Figure 1(b).

In addition, the linear absorption spectra of Ta_2_NiS_5_ are measured. As illustrated in Figure 1(e), the linear absorption also shows apparent anisotropy dependence on the input light polarization. However, the data fluctuation becomes larger after 800 nm. Accordingly, the wavelength of 800 nm with good anisotropy is used for subsequent experiments. The experimental results are plotted as polar diagram shown in Figure 1(f) for 800 nm. It can be easily found that the linear absorption reaches maximum (minimum) when the polarization of the input light is parallel to *a* axis (*c* axis) of Ta_2_NiS_5_ flake.

**Figure 2:**
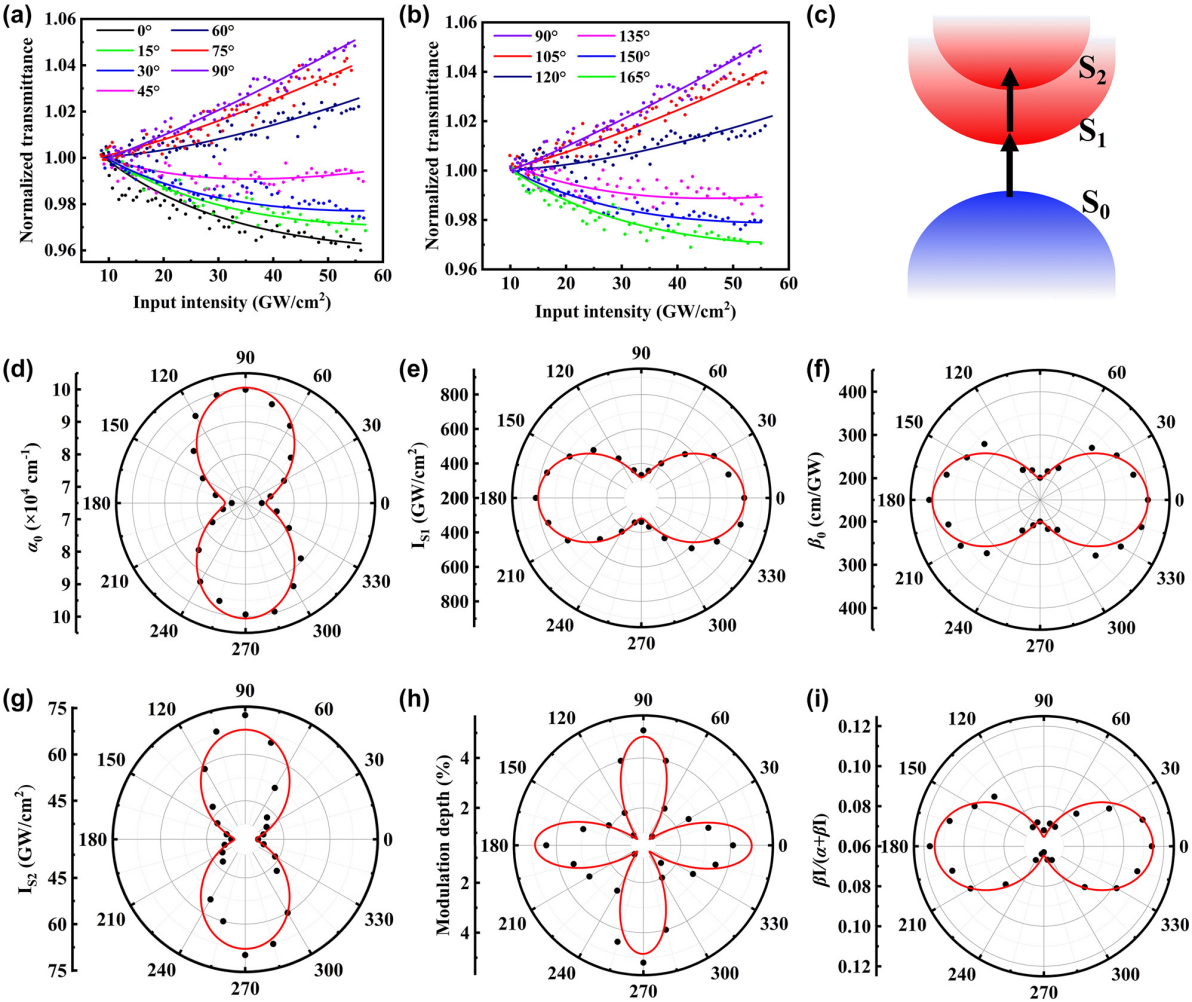
The NLO absorption results of Ta_2_NiS_5 _flake. (a, b) The experimental results of polarization-dependent NLO responses from 0° to 165°. (c) Schematic of NLO absorption in Ta_2_NiS_5_. (d) Linear absorption coefficient, (e) saturation intensity of SA, (f) ESA coefficient, and (g) saturation intensity of ESA versus polarization angle of input laser in polar coordinates. (h) Polar diagram of the anisotropic modulation depth. (i) The dependence of *k* on polarization angle representing the proportion of ESA in the overall NLO absorption process.

Next, we focus on the anisotropic NLO absorption of Ta_2_NiS_5_. The NLO parameters can be obtained from *I*-scan system by measuring the transmittance of material as a function of incident laser intensity. In this system, the Glan-Taylor prism and 1/2 *λ* wave plate were employed to vary the input intensity [39] with the spot size of laser constant, and another 1/2 *λ* wave plate was used to change the polarization direction of input laser. Further, we improved the *I*-scan technique with microscopic imaging (*μ*-*I*-scan) [40] to monitor whether the sample has been damaged by the laser. As a result, the polarization-dependent *μ*-*I*-scan system was utilized to study the anisotropic NLO absorption of Ta_2_NiS_5._ In the experiment, the angle between the polarization direction of incident laser and the *c* axis of Ta_2_NiS_5_ flake was set as *θ* in Figure 1(b), which can be changed from 0° to 345° in 15° a step. As shown in Figure 2(a), when the input intensity increases, the normalized transmittance decreases which indicates a reverse saturable absorption (RSA) response in the Ta_2_NiS_5_ flake with the *θ* in the range of 0°–45°. When the *θ* continuously increases, it can be seen that the normalized transmittance increases, which corresponds to SA response.

For SA effect, it can be explained by the excitation state band-filling effect [41], [42]. As valence band electrons absorb photons and transition into the conduction band, the electrons available for excitation are reduced, and the conduction band is gradually occupied, which increases the transmittance of the sample. For the mechanism of the RSA signal, it may originate from different situations such as two-photon absorption (TPA), free carrier absorption (FCA), or ESA [43], [44]. There are certain conditions for the occurrence of TPA, which usually require an appropriate bandgap and high input intensity. In Figure 2(a) and (b), the RSA signals appear at low input intensity, therefore the TPA effect is not recognized. FCA is an intraband transition with the momentum change of excited carriers. In general, the momentum carried by photons is small. In order to conserve the momentum, FCA needs assistance from phonons or impurities [44]. The transition efficiency of multiparticle processes is intrinsically low. Considering the above factors, the FCA can be safely eliminated. Therefore, ESA is temporarily considered as the cause of this phenomenon. In this process, the electrons of the low-energy excited state further absorb photons and transition to high-energy excited state without momentum change [45], as shown in Figure 2(c). Compared to TPA, this effect is a one-photon absorption, which decreases the transmittance showing the opposite trend to the SA. And, Ta_2_NiS_5_ possesses a direct bandgap and multiple excited states [26], which provide favorable conditions for ESA effect. On the other hand, if the laser pulse width is less than the relaxation time of excited carriers, it is possible for them to transition from S_1_ to S_2_ with sufficient time. In our experiments, the pulse width of femtosecond laser is about 220 fs, and the recombination time of Ta_2_NiS_5_ studied in this work is much longer than the laser pulse width. As a result, the RSA can be reasonably attributed to ESA.

According to the Beer–Lambert principle, after laser passes through an optical medium with a distance of *z*, the attenuation of laser intensity *I* changes as follows,

(4) $$\frac{\text{d}I}{dz}=-\alpha \left(I\right)I$$

where *α*(*I*) represents the intensity-dependent absorption coefficient. Here it is necessary to consider both SA and ESA, then the absorption coefficient is expressed as [41], [45],

(5) $$\alpha \left(I\right)=\alpha +\beta I=\frac{\alpha_{0}}{1+I/I_{S1}}+\frac{\beta_{0}I}{1+{\left(I/I_{S2}\right)}^{2}}$$

where *α*_0_ and *β*_0_ are the linear absorption and ESA coefficients, *I*_*S*1_ and *I*_*S*2_ are the saturation intensities for SA and ESA, respectively. Taking Equations (4) and (5) into consideration, the intensity-dependent transmittance *T* with the distance *L* can be expressed as [45], [46],

(6) $$T\left(I\right)=\exp\left(-\left(\frac{\alpha_{0}}{1+I/I_{S1}}+\frac{\beta_{0}I}{1+{\left(I/I_{S2}\right)}^{2}}\right)L\right)$$

and, the NLO absorption parameters can be extracted by utilizing the Equation (6).

The *I*-scan results at different polarization directions of input laser are shown in Figures 2(a), (b), and S1. The NLO parameters are listed in Table S1, and the values of *α*_0_, *β*_0_, *I*_*S*1_, *I*_*S*2_ are collected and drawn as polar plots shown in Figure 2(d)–(g), where they show good periodicity with the polarization of input laser. In Figure 2(d) and (e), it can be seen that when the polarization of the incident laser is parallel to the *c* axis, the value of *α*_0_ is the smallest, and *I*_*S*1_ reaches the maximum. At the same time, the modulation depth is about 4 % for ESA in Figure 2(h). When the input laser polarization angle aligns with the *a* axis, *β*_0_ and *I*_*S*2_ have the smallest and largest value, respectively, corresponding to a modulation depth of ∼4.9 % for SA. Considering the change of nonlinear mechanism under different polarization directions, we further analyze the proportion of ESA by drawing the coefficient *k* = *βI/*(*α* + *βI*), as shown in Figure 2(i). The ESA takes a larger proportion with higher *k* at 0° indicating that the RSA effect dominated, and the SA effect is importantly crucial at 90°.

To understand the polarization-dependent absorption mechanism, the Fermi’s golden rule is taken into consideration. The transition rate, defined as the probability of the electrons transition from the valance band to the conduction band per unit time, is described as [42],

(7) $$\Gamma_{i\to f}=\frac{2\pi }{\text{h}}{\left|\left\langle f\left|H_{\text{op}}\right|i\right\rangle \right|}^{2}\rho_{f}$$

where $\left\langle f\left|H_{\text{op}}\right|i\right\rangle$ is the transition matrix element of the perturbation *H*_op_ between the initial and final states, and *ρ*_*f*_ is the density of the final states. The matrix element can be calculated by the dipole approximation with an optical transition from the state *i* to *f* [38], [42],

(8) $$\left\langle f\left|H_{\text{op}}\right|i\right\rangle \propto P\cdot D_{fi}$$

where *P* is the polarization vector of the input laser, and *D*_*fi*_ is the dipole vector. The relationship between the optical absorption probability and absorption coefficient *α* is expressed as [38], [42],

(9) $$\alpha \left(E_{L}\right)\propto \underset{f,i}{\sum }{\left|\left\langle f|H_{\text{op}}|i\right\rangle \right|}^{2}\delta \left(E_{f}-E_{i}-E_{L}\right)$$

where *E*_*f*_ is the energy of the electronic state *f*, *E*_*i*_ is the energy of the electronic state *i*, and *E*_*L*_ is the photon energy of the input laser. It can be seen that the absorption coefficient *α* is proportional to the square of the inner product of *P* and *D*_*fi*_. The input laser of different polarization directions can excite interband transition processes with different initial state and final state changing the transition matrix element. Therefore, the absorption coefficient changes with the polarization of the input laser.

Since we have found some clues between the NLO responses and the polarization of input laser, it is meaningful to investigate the polarization-dependent carrier dynamics. And, the response time of Ta_2_NiS_5_-based all-optical devices can be extracted, which is a key factor in constructing the light-control-light system. Therefore, polarization-dependent pump-probe measurement is used to study the anisotropic carrier dynamics of Ta_2_NiS_5_ [42], [47], [48]. In this study, the wavelengths of pump and probe laser are 400 nm and 800 nm, respectively. The polarization angles *θ* of them can be individually changed from 0° to 345° by half wave plate, meanwhile the laser power is constant. The differential transmittance signals Δ*T*/*T*_0_, defined as Δ*T*/*T*_0_ = (*T*−*T*_0_)/*T*_0_, where *T* and *T*_0_ are the probe transmittance of the sample with and without pump laser [49], [50], are collected with the delay time from −50 to 400 ps.

**Figure 3:**
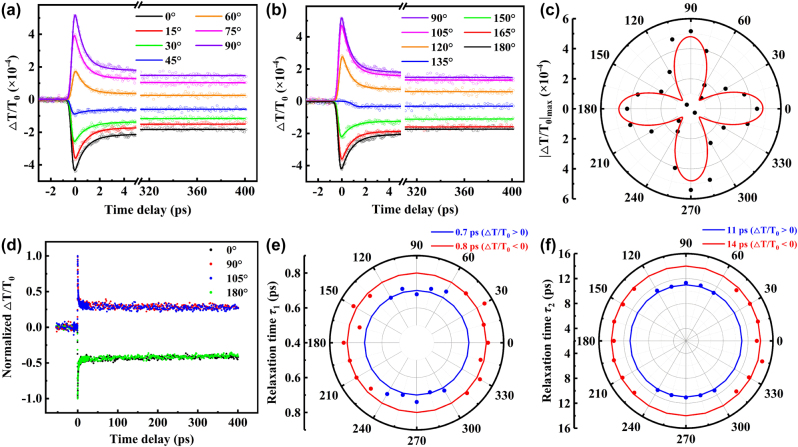
Differential transmittance signal as a function of time delay under different polarization angles of probe laser at (a) 0°–90° and (b) 90°–180° with the pump laser polarization at 0°. (c) Peak signal |Δ*T*/*T*_0_|_max_ with respect to polarization angles of probe laser. (d) The normalized Δ*T*/*T*_0_ as a function of time delay. (e, f) Relaxation time, *τ*_1_ and *τ*_2_, with respect to polarization angles of probe laser.

The modulation effect at different polarization directions of the probe laser on carrier dynamics has been studied and the results are shown in Figure 3, where the pump laser polarization aligns to 0°. The differential transmittance signals exhibit obviously anisotropic with respect to the polarization of probe laser. As the photons of pump laser are absorbed, a large amount of free carriers including free electrons at conduction band and holes at valence band with non-equilibrium state are produced, resulting in the dramatic change of transient transmittance [51]. When the *θ* is rotated between 0° and 45°, the differential transmittance signals are negative, and the amplitude of peaks decrease. The negative peaks indicate an increase in the absorption of probe laser, corresponding to photo-induced absorption. The carriers excited by pump laser can further absorb photons of probe laser and transition to higher energy levels [46], [52]. Interestingly, when the *θ* further increases, the differential transmittance signals change completely. The positive peaks are found, and the amplitude of peaks increase. This phenomenon can be regarded as the band-filling effect. In the same energy level, each quantum state can only consist two electrons with opposite spins. When all the states are filled, the carriers are prevented by the Pauli-blocking effect and the absorption decreases [52]. Therefore, it can be inferred that the ESA dominates when the *θ* is along the *c* axis of Ta_2_NiS_5_, while the band-filling effect plays an important role at *a* axis. The transient absorption processes are consistent with the results of the NLO absorption.

Fitting the differential transmittance data through a bi-exponential decay function, the relaxation time can be obtained. Figures 3(a), (b), and S2 show the fitting results from −3 ps to 400 ps. In our experiments, the photo-induced absorption and band-filling effect are strongest at 0° (180°) and 90° (270°), respectively, resulting in the peak of differential transmittance signal |Δ*T*/*T*_0_|_max_ as a four-petal flower in Figure 3c. Furthermore, the relaxation rate remains unchanged in Figure 3d, which implies that the relaxation process may be isotropic. And, the relaxation times of *τ*_1_ and *τ*_2_ are presented in Figure 3(e), (f), and Table S2. Specifically, the two relaxation time constants, *τ*_1_ and *τ*_2_, are ∼0.8 and 14 ps, respectively, for the photo-induced absorption. And, the lifetime constants of ∼0.7 and 11 ps for fast and slow relaxation processes are obtained from the band-filling effect. It can be seen that the relaxation time constants show little anisotropy. The results demonstrate that the relaxation processes of Ta_2_NiS_5_ are isotropic under the different polarization of probe laser for photo-induced absorption or band-filling process.

**Figure 4:**
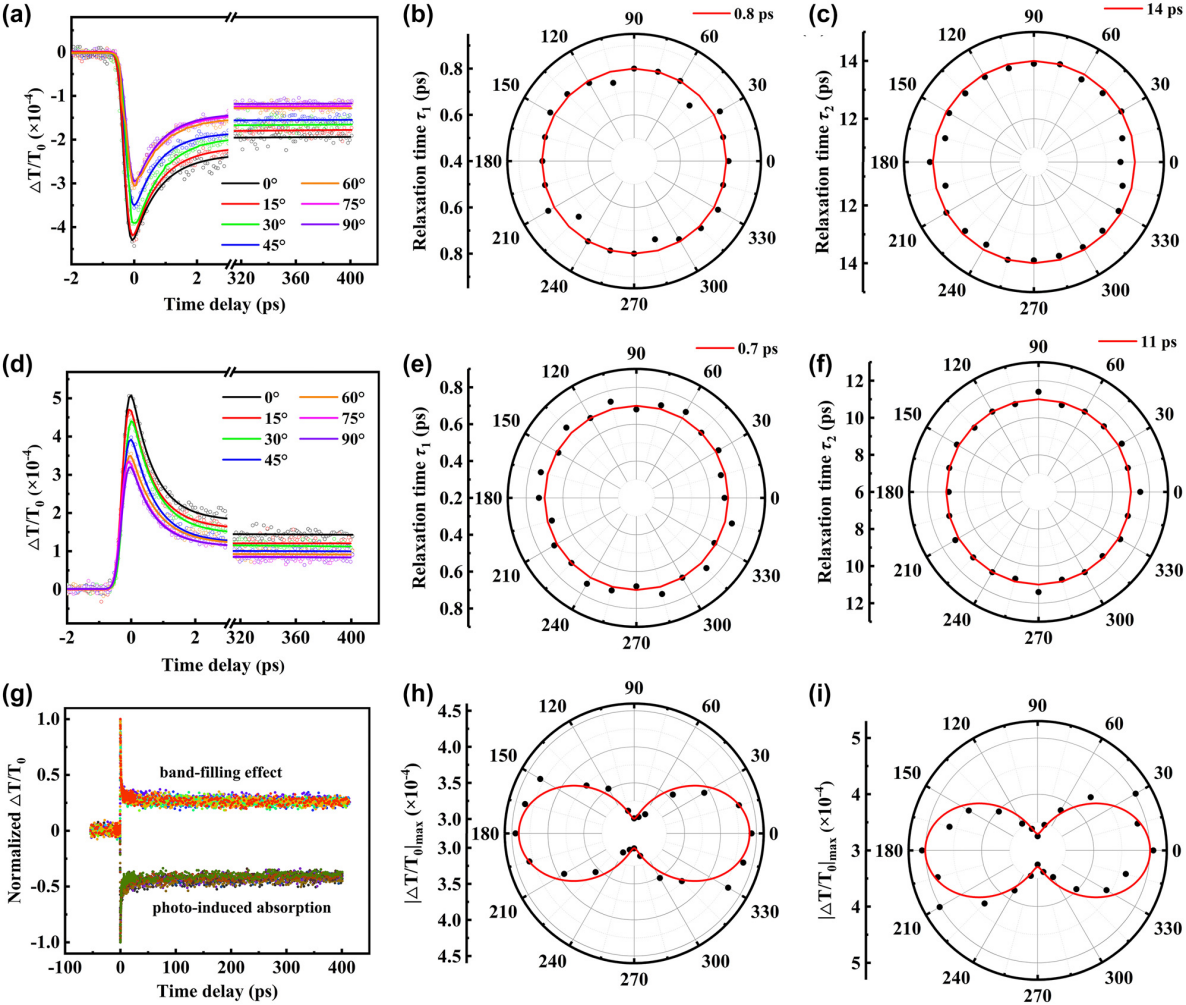
Polarization-dependent pump-probe results. (a) Differential transmittance signal as a function of time delay for different polarization angles of pump laser at 0°–90° with polarization angle of probe laser at 0°. (b, c) Relaxation time *τ*_1_ and *τ*_2_ for photo-induced absorption. (d) Differential transmittance signal as a function of time delay measured for different polarization angles of pump laser at 0°–90° with polarization angle of probe laser at 90°. (e, f) Relaxation time *τ*_1_ and *τ*_2_ for band-filling effect. (g) The normalized Δ*T*/*T*_0_ as a function of time delay from (a) and (d). (h) Peak signal |Δ*T*/*T*_0_|_max_ in (a). (i) Peak signal |Δ*T*/*T*_0_|_max_ in (d).

As mentioned above, we find that the Δ*T*/*T*_0_ signals exhibit anisotropic behavior at different polarization directions of probe laser by ultrafast pump-probe experiments. To investigate the effect about the polarization of the pump laser on carrier dynamics, we modulated the polarization direction of the probe laser to *a* axis or *c* axis and changed the polarization angles of the pump laser from 0° to 180° in a 15° step. When the polarization direction of the probe laser is parallel to the *c* axis, the photo-induced absorption are found, as shown in Figure 4(a). In the range of 0°–90°, the values of |Δ*T*/*T*_0_|_max_ decrease as the polarization angles of the pump laser increase, and opposite trend at 90°–180° is found in Figure S3(a). The band-filling effect does not occur when the polarization of the pump light varies from 0° to 180°. Similarly, the relaxation rate remains unchanged, as shown in Figure 4g. The values of |Δ*T*/*T*_0_|_max_ are different from the dependence about the polarization of the probe laser in Figure 3c, here it has a period with 180° as shown in Figure 4h. Using the biexponential decay function, we obtain the *τ*_1_ and *τ*_2_ are ∼0.8 and 14 ps for photo-induced absorption process in Figure 4(b), (c), and Table S3. The results demonstrated that the relaxation processes are isotropic under different polarization of the pump laser for photo-induced absorption.

Next, we changed the polarization angle of the probe laser to align with *a* axis and repeated the experiments. In Figures 4(d) and S3(c), it can be seen that the transmittance signals remain positive. When the polarization angle of the pump laser gradually increases, the values of |Δ*T*/*T*_0_|_max_ also increase with a period of 180° in Figure 4(i). The same relaxation rate can be found in Figure 4(g) for band-filling effect. As shown in Figure 4(e), (f), and Table S4, the lifetime constants are ∼0.7 and 11 ps for fast and slow relaxation processes, respectively, under different polarization directions of pump laser. In Figure 4, the data from 195° to 345° are repeated from 15° to 165°. In the band-filling process, the Δ*T*/*T*_0_ signals are also anisotropic, and the relaxation processes are isotropic. The relaxation time of photo-induced absorption and band-filling effect under different polarization angles of the pump laser are same as that in Figure 3.

To sum up, we showed the influence of pump and probe laser with different polarization directions on the carrier dynamics of Ta_2_NiS_5_. The anisotropic differential transmittance signals and isotropic relaxation processes with the times of ∼0.8 and 14 ps (∼0.7 and 11 ps) for photo-induced absorption (band-filling effect) are found. The time constant *τ*_1_ can be attributed to carrier-phonon scattering of intraband relaxation process, which is shorter than that of various transition metal dichalcogenides [53], [54], [55], [56]. The *τ*_2_ is regarded as the electron-hole recombination via the assistance of trap states originating from the S vacancy [13]. These results indicate Ta_2_NiS_5_ has potential applications in ultrafast optoelectronic devices.

**Figure 5:**
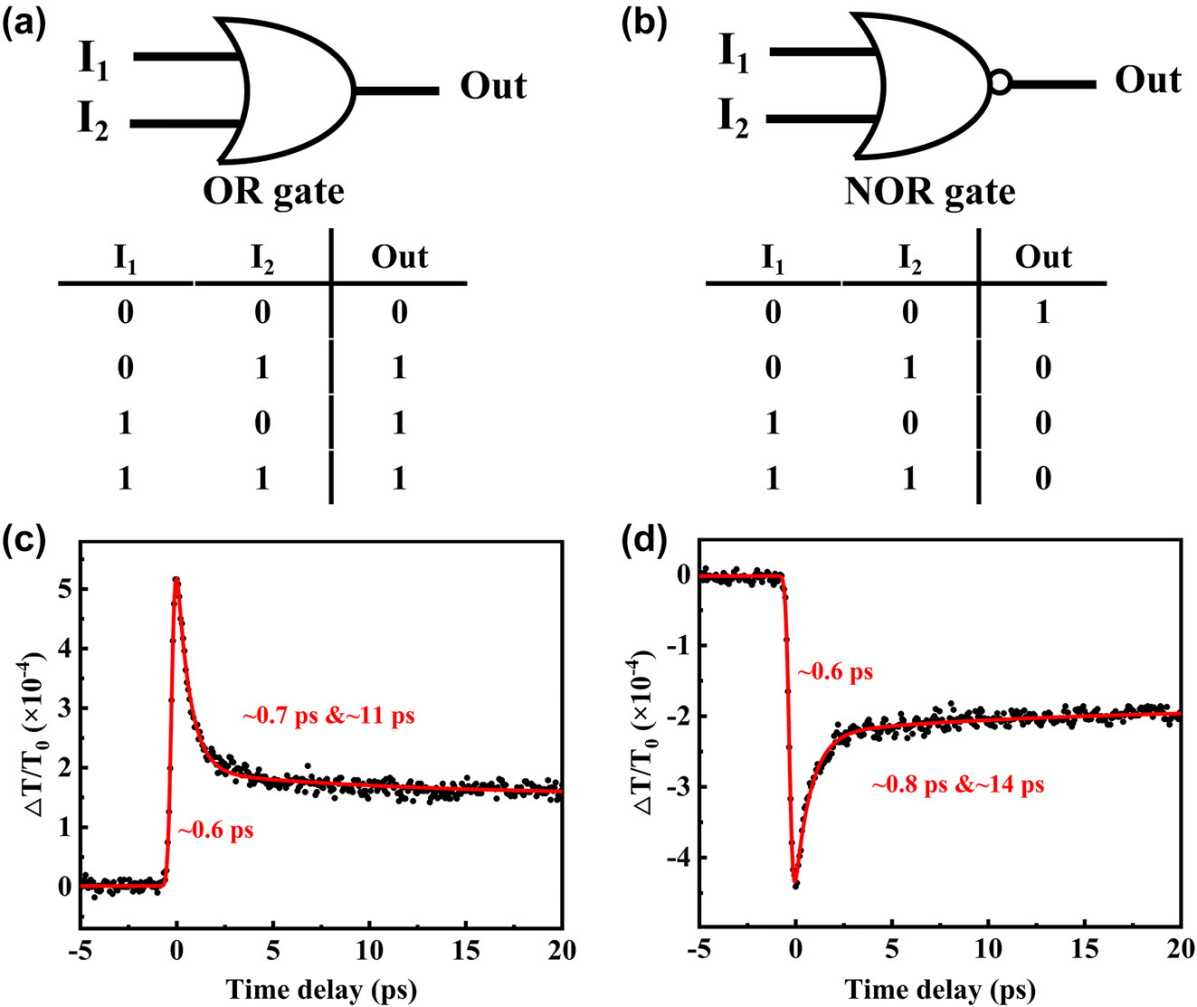
The results of ultrafast all-optical logic gates. (a, b) Schematic and truth table of OR and NOR logic gates. (c, d) The response and recovery times of logic gates extracted from differential transmittance signal in Figure 4.

Based on the polarization-dependent NLO responses and carrier dynamics, we have designed the ultrafast all-optical logic gates utilizing Ta_2_NiS_5_ flake. Two laser pulses with the polarization directions can be individually manipulated as inputs and the laser passing through the sample is regarded as the output terminal. Specifically, the polarization directions of the two input beams (*I*_1_ = *I*_2_ = 55 GW/cm^2^) are modulated along *a* axis of Ta_2_NiS_5_ flake. When at least one laser pulse is on (logical state 1), the transmittance through the sample is enhanced and the output result is positive (logical state 1). As shown in Figure 5(a), the truth table lists the details, which is an all-optical OR logic gate. Through the study of ultrafast carrier dynamics, we can extract the response speed of all-optical logic gate. Figure 5(c) shows the transient transmittance result for band-filling effect revealing an ultrafast response time (*T*_re_) of ∼0.6 ps and a recovery process with a fast (slow) relaxation time of ∼0.7 ps (∼11 ps). In addition, an all-optical NOR logic gate is proposed when the polarization direction of incident laser at 0° in Figure 5(b). When any input laser is on (logical state 1), the transmittance is suppressed due to the ESA, and the output result is negative (logical state 0). Similarly, in Figure 5(d), the response time and recovery time of NOR gate can be extracted. The Ta_2_NiS_5_-based all-optical switches exhibit ultrafast response speed, calculated by 1/*T*_re_ [10], [57], about 1.7 THz and stable performance during a long time duration in our study.

**Figure 6:**
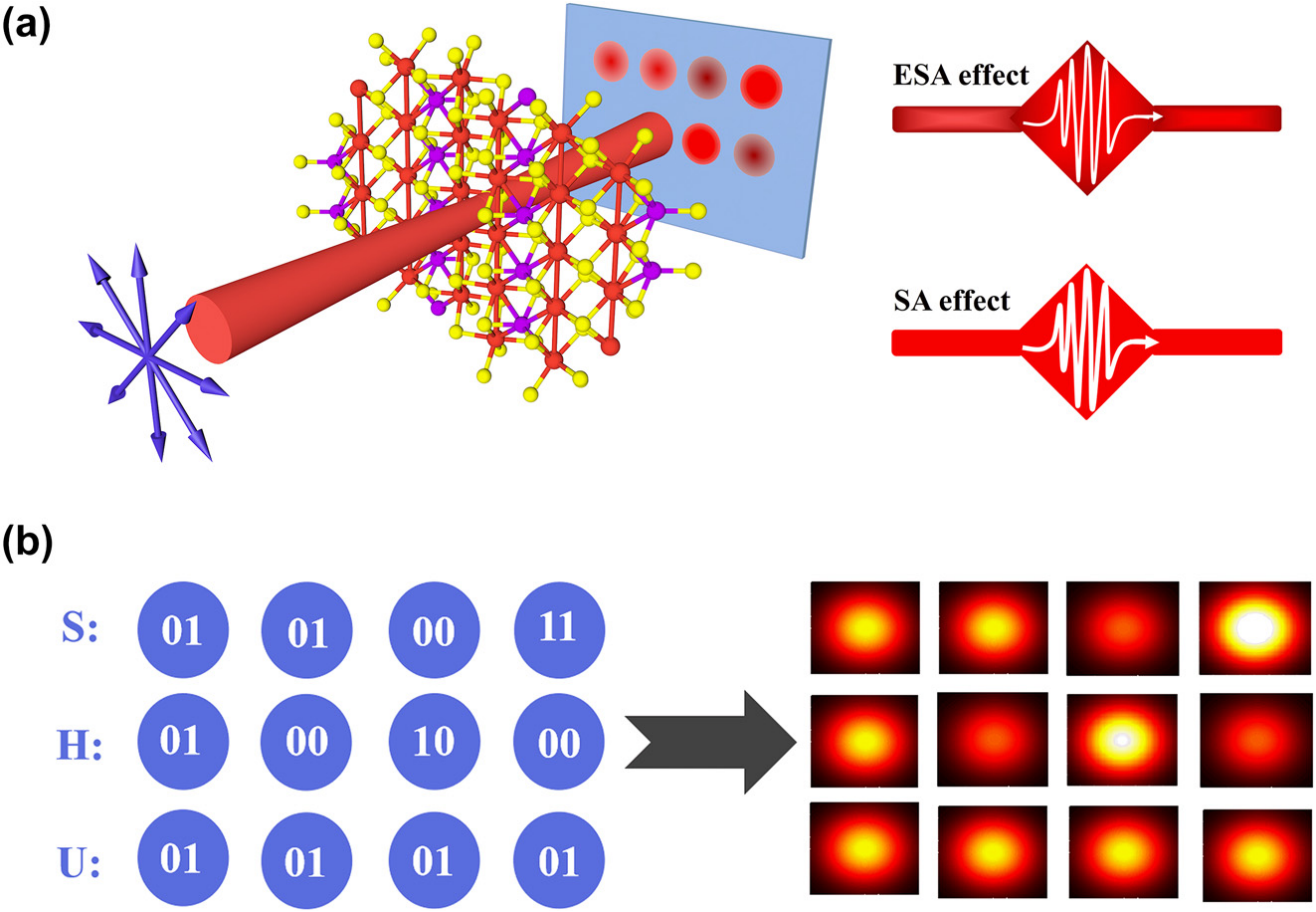
The demonstration of all-optical information transmission. (a) The experimental schematic for optical information transmission. (b) The results of “SHU” encoded at specific intensity and polarization.

We further propose a secure information transmission method using the transmittance of incident laser as the guidance and set the encoding rule by the anisotropic NLO effects. As shown in Figure 6(a), the incident laser of 55 GW/cm^2^ with different polarization directions can excite various NLO responses. The incident laser with specific polarization directions is selected for encoding, corresponding to a unique spot or transmittance as shown in Table 1. The *T*_0_ is the linear transmittance of sample, and *T*_min_ (*T*_max_) represents the minimum (maximum) transmittance of ESA (SA) effect. According to binary codes, an 8-bit binary code sequence can replace a character. By looking up American Standard Code for Information Interchange, the unique encoding can be found for any character, e.g., “0100 0001” for the letter “A”. Therefore, “SHU” (acronym of Shanghai University) can be represented by a 3 × 4 unique optical array, as shown in Figure 6(b). From the NLO responses, we can find that the transmittance is closely dependent on the polarization angle and power of the incident laser. When the laser with random power or mismatched polarization angle is input, an incorrect optical array with meaningless information will be obtained. Only by strictly following the incident laser states listed in Table 1 can the true information be accurately acquired. Therefore, the polarization direction and intensity of incident laser are irreplaceable, they are the key to output the real information. This method based on anisotropic NLO responses of Ta_2_NiS_5_ can prominently enhance the security and accuracy of information transmission. In this study, the Ta_2_NiS_5_ is a layered micro-sample. It is very convenient for potential application in ultrafast optical integration devices such as optical chips and flat panel display devices.

**Table 1:** The encoding rule at specific polarization directions of incident laser using for information transmission.

Polarization direction	Transmittance	NLO response	Encoding	Schematic diagram of spot
0°	*T* = *T*_min_	ESA	“00”	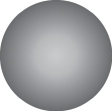
45°	*T*_min_ < *T* < *T*_0_	ESA	“01”	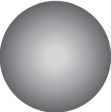
60°	*T*_0_ < *T* < *T*_max_	SA	“10”	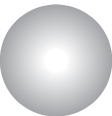
90°	*T* = *T*_max_	SA	“11”	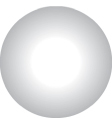

## 3 Conclusions

In summary, we have performed polarization-dependent *I*-scan and pump-probe measurements for anisotropic Ta_2_NiS_5_ flake. Specifically, the crystal axis orientations are identified by polarized Raman spectroscopy, and the anisotropic linear absorption provides a reference of wavelength in experiments. The NLO responses undergo a transition from ESA to SA by changing the polarization directions of incident laser, which is attributed to the anisotropic optical transition probability. In pump-probe experiments, the signals of photo-induced absorption and band-filling effect exhibit anisotropic at different polarization directions of probe or pump pulse with the isotropic relaxation process. Utilizing the unique NLO responses of Ta_2_NiS_5_, we have realized all-optical logic OR and NOR gates with ultrafast response speed of ∼1.7 THz. Additionally, a novel coding method has been proposed to transmit information with higher security and accuracy by manipulating the intensity and polarization of input laser. These results provide extraordinary inspiration for designing Ta_2_NiS_5_-based optoelectronic devices and exploring valuable applications.

## Supplementary Material

Supplementary Material Details
