# Supplementary material for: Anisotropic nonlinear optical responses of Ta2NiS5 flake towards ultrafast logic gates and secure all-optical information transmission
Source: Nanophotonics. 2024 Oct 23;13(24):4429–39. doi: 10.1515/nanoph-2024-0404 (PMC11636461; doi:10.1515/nanoph-2024-0404)
Supplement: Supplementary file 1 — Supplementary Material Details [file j_nanoph-2024-0404_suppl_001.docx]

Supporting Information

**Anisotropic Nonlinear Optical Responses of Ta_2_NiS_5_ Flake towards Ultrafast Logic Gates and Secure All-Optical Information Transmission**

Lei Yan^1^, Ziyao Gong^1^, Qinyong He^1^, Dechao Shen^1^, Anping Ge^2,3^, Ye Dai^1^, Guohong Ma^1^, Liaoxin Sun^2,3,*^, and Saifeng Zhang^1,#^

^1^Department of Physics, Shanghai University, Shanghai 200444, China

^2^State Key Laboratory of Infrared Physics, Shanghai Institute of Technical Physics, Chinese Academy of Sciences, Shanghai 200083, China

^3^University of Chinese Academy of Sciences, Beijing 100049, China

^#^[sfzhang@shu.edu.cn](mailto:sfzhang@shu.edu.cn)

^*^[sunlx@mail.sitp.ac.cn](mailto:sunlx@mail.sitp.ac.cn)


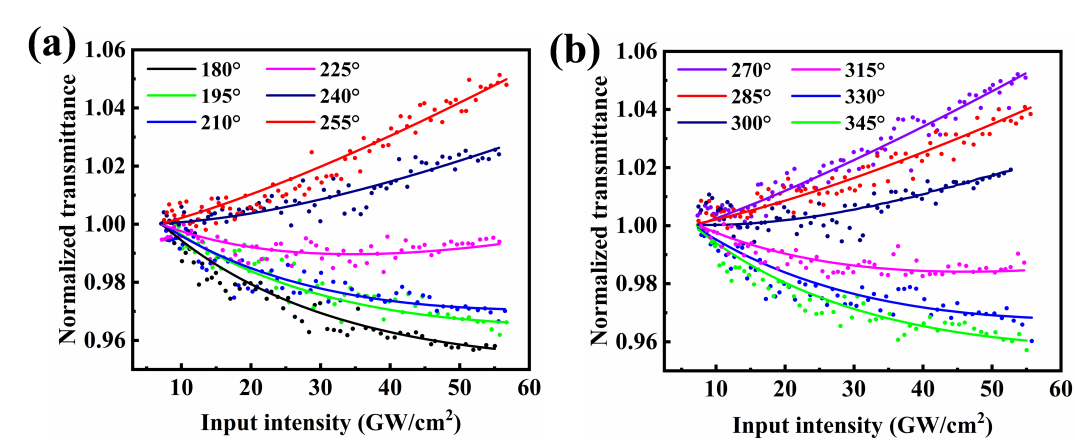


**Figure S1:** The experimental results of polarization-dependent NLO responses from (a) 180° to 255° and (b) 270° to 345°.

**Table S1:** Polarization-dependent nonlinear optical absorption parameters of Ta_2_NiS_5_.

| Polarization angle | *α_0_*  (cm^-1^) | *I_S1_*  (GW/cm^2^) | *β_0_*  (cm/GW) | *I_S2_*  (GW/cm^2^) |
| --- | --- | --- | --- | --- |
| 0° | 70017 | 798.4 | 401 | 36.7 |
| 15° | 72972 | 724.7 | 373.8 | 38.6 |
| 30° | 78717 | 686.8 | 355 | 40.5 |
| 45° | 84786 | 559.3 | 320 | 42.5 |
| 60° | 92467 | 431.7 | 235 | 51.7 |
| 75° | 96508 | 362.6 | 218.2 | 64.9 |
| 90° | 99916 | 333 | 201 | 72.7 |
| 105° | 99337 | 366.4 | 220.5 | 68.6 |
| 120° | 95956 | 464.2 | 229.8 | 58.8 |
| 135° | 87661 | 592.5 | 332.6 | 47.4 |
| 150° | 80242 | 680.5 | 345.9 | 42.9 |
| 165° | 74537 | 766.4 | 374.4 | 38.7 |
| 180° | 69260 | 812.9 | 407 | 37 |
| 195° | 72202 | 759 | 370 | 39.4 |
| 210° | 76806 | 693 | 362.3 | 41 |
| 225° | 85494 | 539 | 324.7 | 42.7 |
| 240° | 92993 | 426 | 232 | 55 |
| 255° | 96231 | 348 | 211 | 60 |
| 270° | 99337 | 339 | 200 | 70 |
| 285° | 99626 | 373 | 219.7 | 67.6 |
| 300° | 94594 | 470 | 230 | 60 |
| 315° | 89144 | 613.6 | 332 | 47 |
| 330° | 80462 | 707 | 366 | 43.7 |
| 345° | 74944 | 797 | 393 | 38.7 |


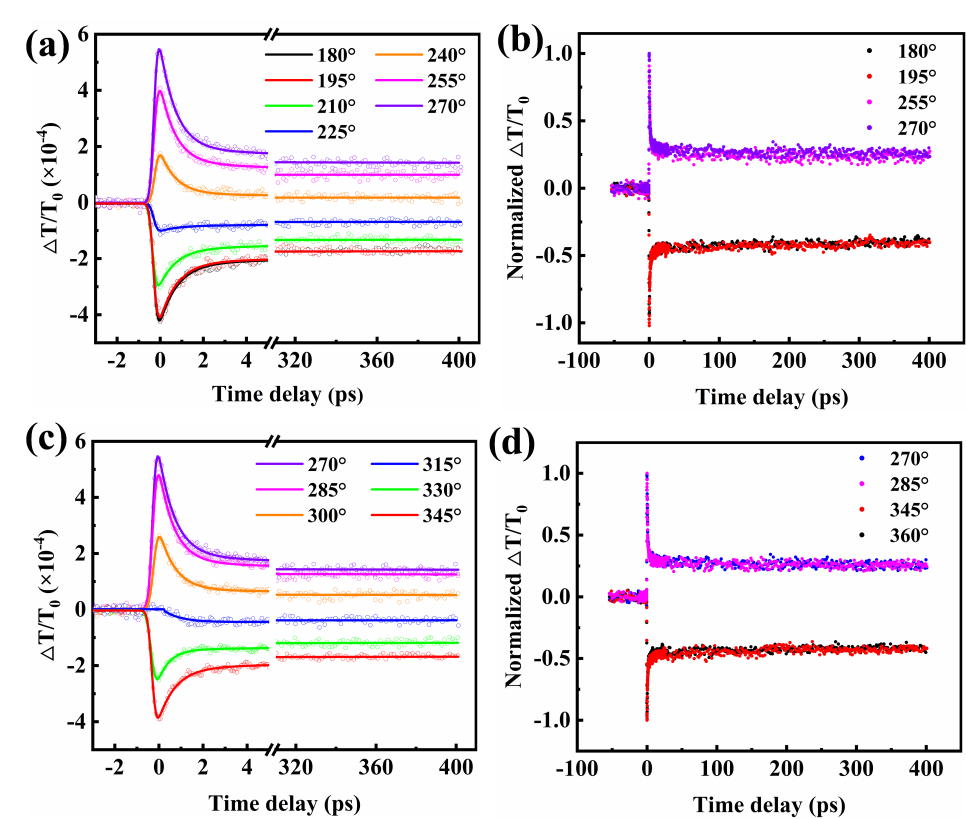


**Figure S2:** (a, c) Differential transmission signal as a function of time delay under different polarization angles of probe laser at 180° to 270°, and 270° to 345° and pump laser at 0°. (b, d) The normalized ΔT/T_0_ as a function of time delay corresponding to (a) and (c).

**Table S2:** The relaxation times of *τ*_1_ and *τ*_2_ at different polarization directions of probe laser.

| Polarization angle | Relaxation time  *τ*_1_ (ps) | Relaxation time  *τ*_2_ (ps) |
| --- | --- | --- |
| 0° | 0.81 | 13.4 |
| 15° | 0.8 | 14 |
| 30° | 0.85 | 13.9 |
| 45° | 0.8 | 13.5 |
| 60° | 0.74 | 10.9 |
| 75° | 0.72 | 11.2 |
| 90° | 0.68 | 11.3 |
| 105° | 0.72 | 11 |
| 120° | 0.7 | 11.3 |
| 135° | 0.78 | 13.8 |
| 150° | 0.82 | 13.7 |
| 165° | 0.74 | 14 |
| 180° | 0.82 | 13.6 |
| 195° | 0.9 | 14 |
| 210° | 0.8 | 13.8 |
| 225° | 0.77 | 13.8 |
| 240° | 0.74 | 11.1 |
| 255° | 0.71 | 11 |
| 270° | 0.74 | 11.1 |
| 285° | 0.69 | 11 |
| 300° | 0.72 | 11.2 |
| 315° | 0.81 | 13.5 |
| 330° | 0.82 | 13.7 |
| 345° | 0.78 | 14.7 |


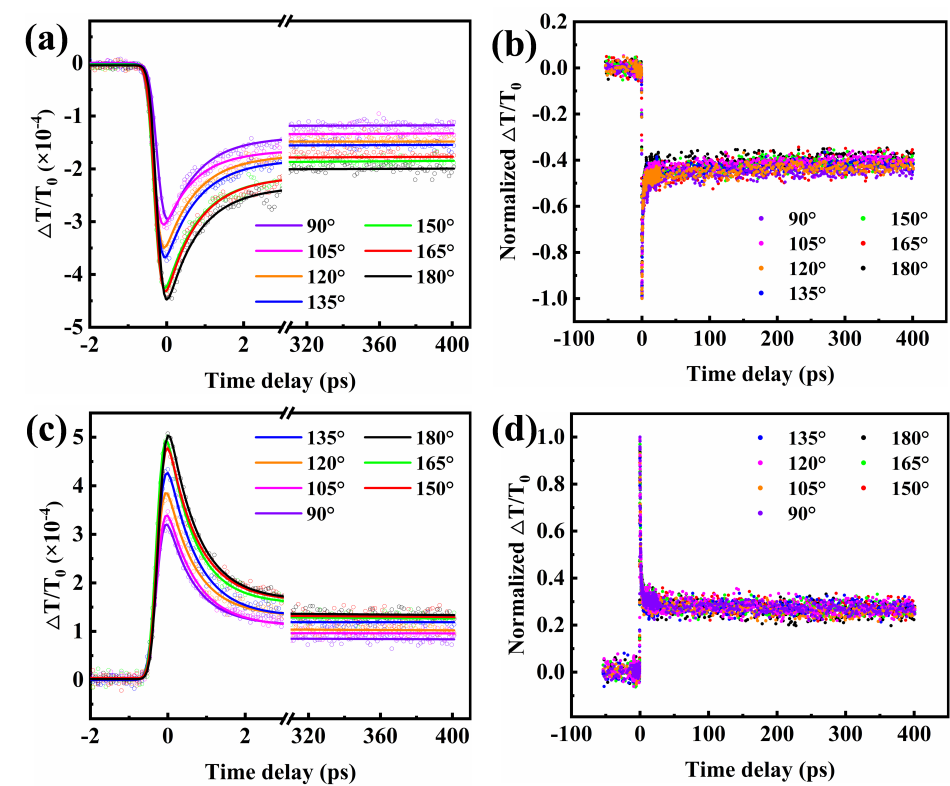


**Figure S3:** (a, c) Differential transmission signal as a function of time delay for different polarization angles of pump laser at 90° to 180° with polarization angle of probe laser at 0° and 90. (b, d) The normalized ΔT/T_0_ as a function of time delay corresponding to (a) and (c).

**Table S3:** The relaxation times of *τ*_1_ and *τ*_2_ at different polarization angles of pump laser with polarization angle of probe laser at 0°.

| Polarization angle | Relaxation time  *τ*_1_ (ps) | Relaxation time  *τ*_2_ (ps) |
| --- | --- | --- |
| 0° | 0.81 | 13.5 |
| 15° | 0.8 | 13.7 |
| 30° | 0.83 | 14 |
| 45° | 0.74 | 13.9 |
| 60° | 0.8 | 13.8 |
| 75° | 0.8 | 14 |
| 90° | 0.8 | 13.9 |
| 105° | 0.75 | 13.9 |
| 120° | 0.79 | 13.9 |
| 135° | 0.81 | 13.9 |
| 150° | 0.82 | 13.9 |
| 165° | 0.8 | 13.7 |
| 180° | 0.8 | 14.1 |

**Table S4:** The relaxation times of *τ*_1_ and *τ*_2_ at different polarization angles of pump laser with polarization angle of probe laser at 90°.

| Polarization angle | Relaxation time  *τ*_1_ (ps) | Relaxation time  *τ*_2_ (ps) |
| --- | --- | --- |
| 0° | 0.68 | 11.5 |
| 15° | 0.68 | 11 |
| 30° | 0.71 | 11.2 |
| 45° | 0.7 | 11 |
| 60° | 0.73 | 11 |
| 75° | 0.72 | 11 |
| 90° | 0.68 | 11.4 |
| 105° | 0.74 | 10.9 |
| 120° | 0.7 | 11 |
| 135° | 0.74 | 10.9 |
| 150° | 0.69 | 11.1 |
| 165° | 0.73 | 11 |
| 180° | 0.73 | 10.9 |
